# Development and Performance of Bioactive Compounds-Loaded Cellulose/Collagen/Polyurethane Materials

**DOI:** 10.3390/polym12051191

**Published:** 2020-05-23

**Authors:** Iuliana Spiridon, Narcis Anghel, Maria Valentina Dinu, Stelian Vlad, Adrian Bele, Bianca Iulia Ciubotaru, Liliana Verestiuc, Daniela Pamfil

**Affiliations:** 1 “Petru Poni” Institute of Macromolecular Chemistry, Grigore Ghica–Vodă 41, 700487 Iași, Romania; spiridon@icmpp.ro (I.S.); vdinu@icmpp.ro (M.V.D.); vladus@icmpp.ro (S.V.); bele.adrian@icmpp.ro (A.B.); pamfil.daniela@icmpp.ro (D.P.); 2 Faculty of Medical Bioengineering, Grigore T. Popa University of Medicine and Pharmacy, 9-13 Kogălniceanu Street, 700454 Iași, Romania; ciubotaru.bianca@icmpp.ro (B.I.C.); liliana.verestiuc@bioinginerie.ro (L.V.)

**Keywords:** cellulose, collagen, biomaterials, tannins, lipoic acid, *Quercus robur* L.

## Abstract

Here we present a new biomaterial based on cellulose, collagen and polyurethane, obtained by dissolving in butyl imidazole chloride. This material served as a matrix for the incorporation of tannin and lipoic acid, as well as bioactive substances with antioxidant properties. The introduction of these bioactive principles into the base matrix led to an increase of the compressive strength in the range 105–139 kPa. An increase of 29.85% of the mucoadhesiveness of the film containing tannin, as compared to the reference, prolongs the bioavailability of the active substance; a fact also demonstrated by the controlled release studies. The presence of bioactive principles, as well as tannins and lipoic acid, gives biomaterials an antioxidant capacity on average 40%–50% higher compared to the base matrix. The results of the tests of the mechanical resistance, mucoadhesiveness, bioadhesiveness, water absorption and antioxidant capacity of active principles recommend these biomaterials for the manufacture of cosmetic masks or patches.

## 1. Introduction

Natural polymers present a large variety of biological applications due to their low cost, biodegradability and biocompatibility, and have become, in recent years, an important starting point for biomaterials with applications in medicine, as delivery systems for drugs and cell therapies, or as scaffolds for tissue engineering, implants and wound dressings [1]. It is well known that controlled biodegradability and structural integrity in physiological conditions are very important properties for improved biomaterials. Herein, an environmentally acceptable and recyclable solvent [2], namely 1-(n-Butyl)-3-methylimidazolium chloride, was used to solubilize cellulose, collagen and polyurethane. Some studies reported that ionic liquids could enhance the transdermal absorption of drugs [3,4].

Cellulose is the most abundant semi-crystalline natural polymer, consisting of repeating glucose units bounded by β-1,4-glycosidic bonds [5]. It presents a good hydrophilicity, high sorption capacity and cost-effectiveness, as well as biocompatibility and an ability to maintain moisture, which recommend cellulose for different biomedical or cosmetic applications [6].

Cellulose and its derivatives have found wide applications in various fields. Thus, carboxycellulose has been shown to be effective as a hemostatic, being used in surgical sutures [7]. The incorporation of titanium dioxide-like pigments into the structure of nanocrystalline cellulose has not only increased its resistance to paint degradation, but has also imparted antibacterial properties [8]. Nanocellulose has also paved the way for the design of environmentally friendly, biocompatible materials, that have proven effective as retention agents for heavy metals [9,10,11,12,13]. Cellulose esters have been shown to have good thermoplastic properties and, moreover, have been developed as compatibilizers and reinforcing agents with other polymers [14]. The development of nanostructured cellulose-based structures has expanded the area of use of this biopolymer in wastewater treatment [15,16], the stabilization of carbon nanotubes [17], and the development of new composites for drug transport [18].

Collagen is the most abundant protein in animals, and constitutes the matrix of skin, bones and other tissues. We have considered collagen type I as a component of our biomaterials because it is a triple-helical conformation comprising of three polypeptide chains intertwined in a right-handed manner, and it is one of the main components of the extracellular matrix. It has a fibrillar morphology [19] and exhibits elasticity and mechanical toughness [20]. Some studies demonstrate that the incorporation of substances from the category of flavonoids in the protein matrix of collagen reduces the susceptibility of the latter to oxidative stress, as showed by Lucarini et al. [21]. Cellulose–collagen composites have been shown to have good mechanical properties, which is vital for practical application. Such bio composites have been used successfully as scaffold material in tissue engineering [22,23]. Moreover, the biocompatibility of cellulose and collagen with the human body allowed the design of matrices with an osteogenic effect on mesenchymal stem cells [24].

Polyurethanes have attracted attention for their potential use in medical applications, especially when they are functionalized using different natural compounds [25]. Polyurethanes composites are used as medical implants, such as cardiac pacemakers and vascular grafts, and due to excellent mechanical properties and biocompatibility, they could be used in regenerative medicine. The introduction of microcrystalline cellulose in the base matrix of polyurethane elastomers has resulted in an increase in the mechanical strength properties of the material in question, as well as in the thermal stability [26,27].

These above-mentioned components were chosen due to the importance of the toxicity, safety and environmental compatibility of biomaterials for various applications.

At the same time, the incorporation of different biological agents into biomaterials, and their controlled release, represents a proper way to control different processes such as inflammation, infections or stimulation of tissue regeneration [28]. Different Quercus species have been shown to possess antimicrobial, anti-inflammatory, gastroprotective, hemolytic and antioxidant properties [29]. Since ancient times, these species have been used to treat inflammation diseases, tannins being widely distributed in their compositions. Tannins are plant-based substances which belong to the polyphenols’ class (from the polyphenolcarboxylic acid series, or from the phenyl-benzopyran series). Tannins are highly astringent, precipitating substances of a protein nature. At the same time as the coagulation of proteins, there is also an action of retraction of the tissue, thus reducing the action surface, a property that is used to treat wounds.

Lipoic acid is a natural antioxidant compound and an oxidative stress scavenger, and has been used as a drug carrier for pathological conditions characterized by oxidative stress, including cancer and neurodegenerative diseases [30], and also as an anti-inflammatory agent [31]. It is a hydrophobic substance derived from caprylic acid, and contains two sulfur atoms connected by a disulfide bond, which is thus considered to be oxidized [32].

In the light of fact that the skin is the largest organ of the body, in the current study, the addition of lipoic acid and tannin to cellulose–collagen–polyurethane matrix has been studied. The reason for choosing this formulation was the fact that cellulose ensures the mechanical strength of the polymer matrix, polyurethane gives the necessary elasticity for topical application, and collagen gives bioadhesion. Lipoic acid and tannins in oak bark were chosen as bioactive principles due to their antioxidant properties and biocompatibility with the human body.

To our knowledge, until now, no evaluations of cellulose–collagen–polyurethane formulations, comprised of either tannin or lipoic acid, have been reported in the literature. Having in mind that some interactions between the used fillers and matrix could occur, the mucoadhesiveness, the in vitro filler release and the antioxidant activity of the materials were evaluated. The morphology, interactions between components, water sorption capacity and mechanical properties of the materials have also been investigated, by scanning electron microscopy (SEM), Fourier transform infrared spectroscopy (FTIR), Dynamic vapor sorption (DVS) and compression tests.

## 2. Materials and Methods

### 2.1. Materials

Cellulose (cotton linters, ~20 micrometers, 240 Da), collagen hydrolysate, a polypeptide made by further hydrolysis of denatured collagen (molecular weight of 96 kDa; due to semantics, and ease of reading, we use the generic name “collagen” for the rest of article), lipoic acid, gallic and ellagic acids were purchased from Sigma-Aldrich (St. Louis, MO, USA) and used without further purification. Oak bark (*Quercus robur* L.) was obtained from a local drugstore.

### 2.2. Methods

#### 2.2.1. Polyurethane Synthesis

The polyurethanes used in this study were synthesized from polycaprolactone (PCL), methylene diphenyl diisocyanate (MDI) and a mixture of butane diol (BD) and beta-cyclodextrin (β-CD) at a ratio of 9/1 (*w/w*) as chain extender, in dimethylformamide (DMF) solution. Briefly, the PCL was dried in vacuum 1 mm Hg at 80 °C for 3 h. The reaction was carried out under stirring with MDI at the temperature of 80 °C for 1 h and then the DMF solution mixture of BD and β-CD was added and the mass reaction was kept under stirring at 60 °C for 6 h. The polyaddition reaction was stopped with a solution of 5 mL EtOH:DMF 1:1 (*v/v*) at the viscosity of ~7000 cP. Molar ratio between components PCL/MDI/(BD/β-CD) was 3/4/1 [33].

**Scheme 1:**
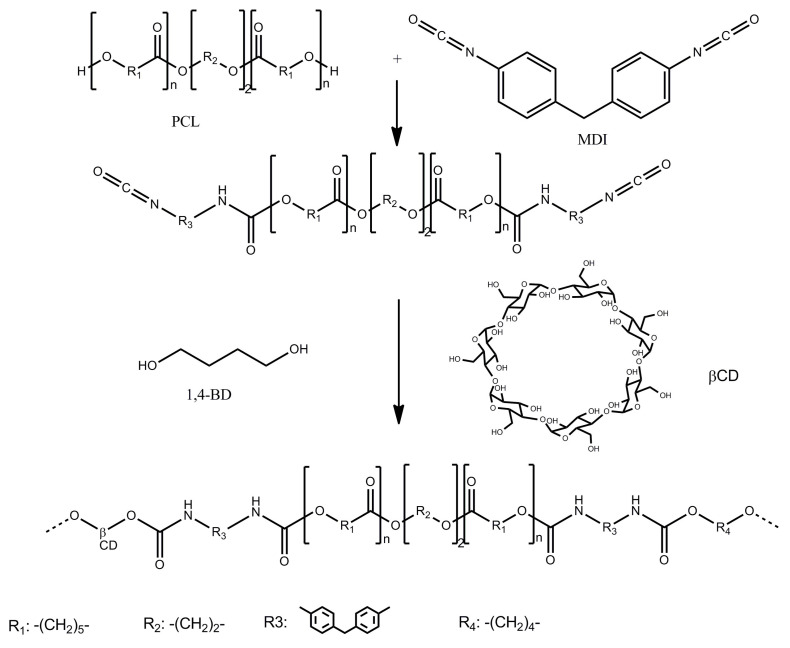
Polyurethane synthesis.

The synthesis route is shown in Scheme 1.

#### 2.2.2. Tannins Extraction

**Scheme 2:**
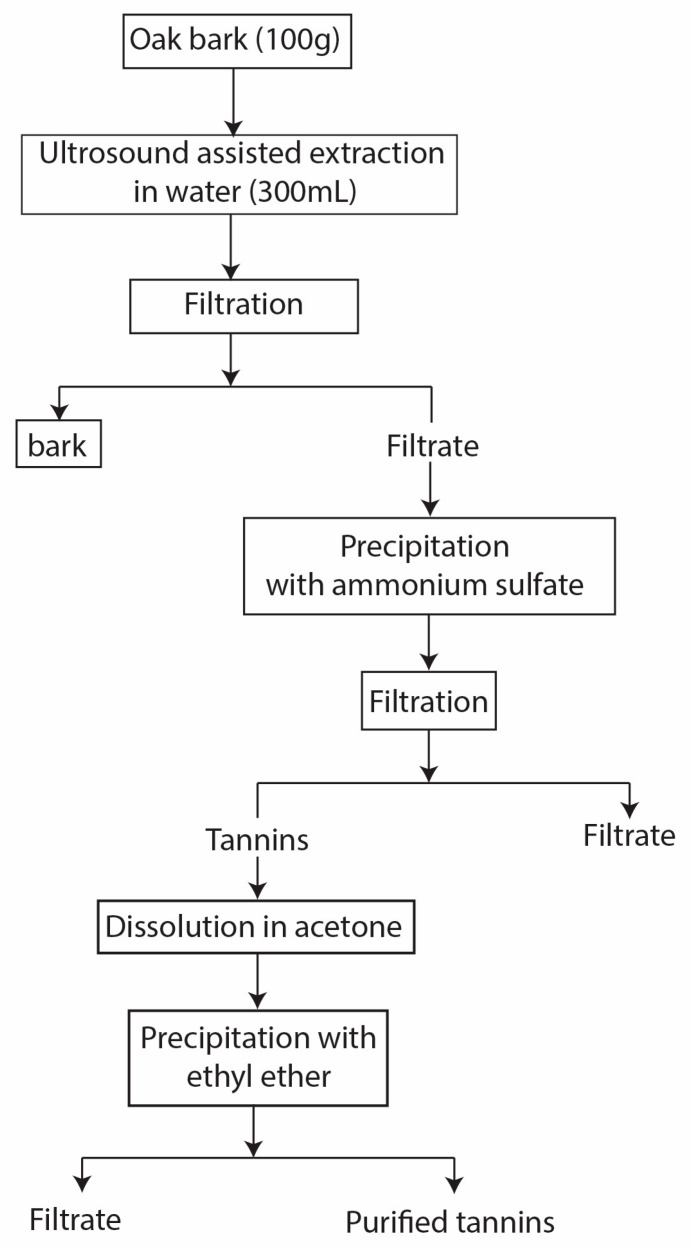
The workflow for the tannin’s extraction.

Tannins extraction was performed according to the method described by Sivakumar et al. [34] with slight modifications (Scheme 2). Oak bark was cut into small sizes in the range of 1 to 2 cm. Then a pre-selected amount of the bark material (100 g) was taken in a clean glass beaker. Extraction was carried out using 300 mL of distilled water. Ultrasound-assisted extraction was carried out twice using a power level of 240 W for 30 min. The aqueous extract was filtered and the tannins were precipitated by saturating the solution with ammonium sulfate. The crude material was subsequently purified by dissolution in acetone and reprecipitation with ethyl ether.

#### 2.2.3. Preparation of Biomaterials

The reference material (REF) was obtained by dissolution of cellulose (1 g), collagen (0.25 g) and polyurethane (0.25 g) in buthyl-3-methylimidazolium chloride (10 g) under stirring, at a temperature of 100 °C for 8 h. Other biomaterials named LIP and TAN were obtained by addition of 0.15 g of lipoic acid and tannin, respectively, to the cellulose–collagen–polyurethane matrix. After 48 h, the samples were washed with distilled water. The respective amounts of tannin and lipoic acid removed from the TAN and LIP formulation were evaluated by Ultraviolet–Visible (UV) spectroscopy. It was found that 4.3% of tannin and 2.8% lipoic acid were removed by washing.

#### 2.2.4. Characterization

##### FTIR Spectroscopy

Fourier Transform Infrared Spectroscopy (FTIR) spectroscopy was used to analyze the possible interaction materials’ components. The film samples were measured by a Bruker, Vertex 70 (Billerica, MA, USA) equipped with an attenuated total reflection (ATR) device. All samples were acquired using a diamond crystal with ZnSe focusing element at room temperature. Scanning was performed in a range from 4000 cm^−1^ to 600 cm^−1^ with a spectral resolution of 2 cm^−1^, with 64 repetitious scans averaged for each spectrum. Prior to measurement, the materials were conditioned at 65% ± 2% relative humidity and 20 ± 2 °C for 48 h.

##### Scanning Electron Microscopy

Scanning Electron Microscopy (SEM) was used to analyze the cross sections of material using a SEM (FEI QUANTA 200ESEM instrument) with an integrated EDX system: GENESIS XM2i EDAX with an SUTW detector. The samples were analyzed with a low-vacuum secondary electron detector at an accelerating voltage of 25.0 kV, at room temperature and 0.050 Torr internal pressure. The experiment was performed in triplicate.

#### 2.2.5. Bioadhesivity Test

A TA.XT plus^®^ analyzer from Stable Micro Systems (Godalming, UK) was used to evaluate the adhesion force (maximum detachment force) and total work of adhesion as described in a previous paper [35]. The bioadhesion test was performed on a hydrated dialysis tubing membrane (cellulose, Visking DTV14000), in PBS (pH 7.4) at 37 °C, while for the determination of mucoadhesive properties a fresh porcine skin membrane was used. The values given for each sample are the results of five determinations.

#### 2.2.6. Compression Test

The compressive properties of the materials were determined using a Shimadzu Testing Machine EZTest (EZ-LX/EZ-SX Series, Kyoto, Japan) at a compression rate of 1 mm × min^−1^. This test was performed at 22 °C and was applied on samples, as plates, with 10 mm thickness, 12 mm width and 4 mm height. The setup of the test and the calculations of the elastic modulus were performed in accordance with the procedure already reported for curdlan-based hydrogels [36]. Compressive strength was determined as the compressive stress at 10% strain, while the elastic modulus was calculated as the slope of the initial linear region in the stress-strain curve.

#### 2.2.7. Dynamic Vapor Sorption (DVS)

IGAsorp equipment (Hiden Analytical, Warrington, UK) was used to evaluate the water sorption at atmospheric pressure by passing a humidified stream of gas over the sample. Isothermal studies were performed at humidity between 0% and 95%, in the temperature range from 5 °C to 85 °C, with an accuracy of ±1% for 0%–90% Relative Humidity (RH) and ±2% for 90%–95% RH.

#### 2.2.8. In Vitro Release Studies

The experiments were carried out in a 708-DS Dissolution Apparatus coupled with a Cary 60 UV-VIS spectrophotometer (Agilent Technologies, Santa Clara, CA, USA) at 37 ± 0.5 °C and a rotation speed of 100 rpm, in media with phosphate buffered saline (pH 7.2). The concentration of the released compound was analyzed spectrophotometrically, showing λ_max_ values of 276 nm (TAN) and 287 nm (LIP) at room temperature. The concentrations were calculated based on the calibration curves determined at the same wavelengths. The filler release kinetics were evaluated using the equation proposed by Korsmeyer and Peppas [Equation (1)] [37].

(1) $$\frac{M_{t}}{M_{\infty }}=kt^{n}$$

where *M_t_*/*M*_∞_ represents the fraction of the drug released at time *t*; *M_t_* and *M*_∞_ are the absolute cumulative amount of drug released at time *t* and the maximum amount released in the experimental conditions used, at the plateau of the release curves; *k* is a constant incorporating the characteristics of the macromolecular drug loaded system, and *n* is the release exponent, which is indicative of the release mechanism.

In the equation above a value of *n* = 0.5 indicates a Fickian diffusion mechanism of the filler from the biomaterial sample, while a value 0.5 < *n* < 1 indicates a non-Fickian behavior. When *n* = 1, a case II transport mechanism is involved with zero order kinetics, while *n* > 1 indicates a special case II transport mechanism [38].

#### 2.2.9. (DPPH—2,2 diphenyl-1-picrylhydrazyl) Assay

The 2,2 diphenyl-1-picrylhydrazyl (DPPH) free radical scavenging activity was determined by the method described by Sridhar and Charles [39] with slight modifications. For the DPPH assay, samples with weights varying between 20 and 70 mg were added to the same volume (10 mL) of DPPH methanolic solution (100 μM). Mixtures were shaken and left to incubate for 20 min in the dark at room temperature. A decrease in absorbance was measured at 515 nm against a blank of methanol without DPPH using a Jenway 6405 UV/Vis spectrophotometer. The inhibition percentage of DPPH discoloration was calculated using Equation (2), where *A_control_* is the absorbance of control and *A_sample_* is the absorbance of the sample.

(2) $$\%inhibition=\left[\frac{A_{control}-A_{sample}}{A_{control}}\right]\times 100$$

Values are expressed as mean ± SD of triplicate measurements.

#### 2.2.10. HPLC Analysis of Tannins

A Shimadzu Prominence High Performance Liquid Chromatography (HPLC) System equipped with an Alltech Econosil C18 column (4.6 × 250 mm, 5 µm) was used for HPLC analysis. Elution: solvent A (water with 1% AcOH), solvent B (methanol). Gradient: solvent B from 20% to 100% in 60 min. Flow rate 1 mL/min. Temperature 25 °C, injection volume 20 µL. UV detection at 280 nm. The tannins were dissolved in the initial mobile phase at a concentration of 1 mg/mL. Gallic and ellagic acids have been used as standards for identifying the components of tannic extract [40]. The compounds were quantified using the calibration curves method [41,42].

## 3. Results and Discussion

The design and characterization of novel biomaterials, obtained without chemical modification, is of great relevance for the development of biomaterials with various applications.

### 3.1. Analysis of Tannins in Oak Bark

**Figure 1:**
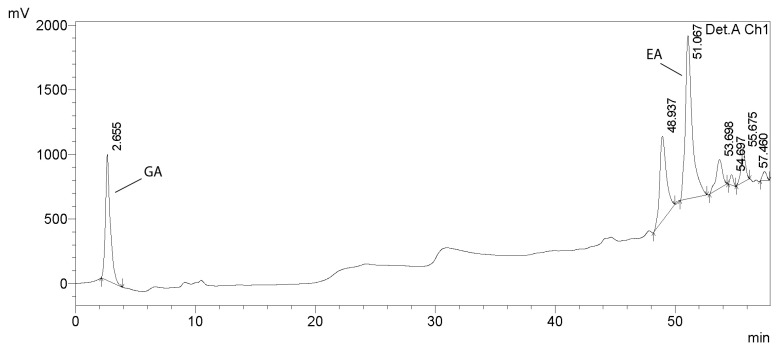
HPLC chromatogram for *Quercus robur* L. extract (GA—gallic acid, EA—ellagic acid).

The chromatogram in Figure 1 indicates the presence of gallic and ellagic acids, predominant in the aqueous extract of the oak species *Quercus robur* L. [41]. Scientific data stipulate a ratio between ellagic and gallic acid of about 10/1 [42]. In this case, the ellagic and gallic acids were found in amounts of 83.3 and 7.2 mg/g extractum pulvis of oak bark.

### 3.2. FTIR Analysis

**Figure 2:**
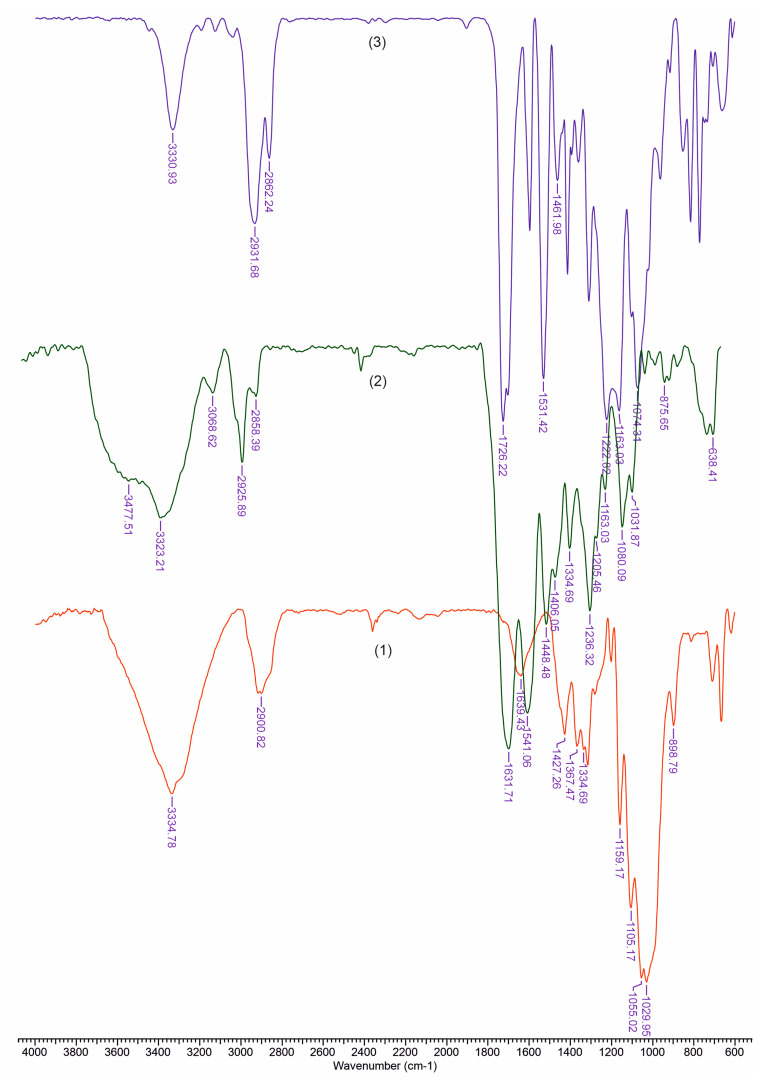
FTIR spectra of reference’ components: (**1**)—cellulose; (**2**)—polyurethane; (**3**)—collagen.

FTIR spectroscopy was used to analyze the possible interactions between material components. Figure 2 depicts the FTIR spectra for components of the reference film. The peaks observed in cellulose spectra [Figure 2(1)], in the range of 3660–2900 cm^−1^, are characteristic for the stretching vibration of O–H and C–H bonds in polysaccharides, while the broad peak at 3334 cm^−1^ is characteristic for the stretching vibration of the hydroxyl group in polysaccharides [43]. The band at 2900 cm^−1^ is attributed to the C–H stretching vibration of all hydrocarbon constituents in polysaccharides. Typical bands assigned to cellulose were observed in the region of 1630–900 cm^−1^. The peaks located at 1631 cm^−1^ correspond to the vibration of water molecules absorbed in cellulose [44]. The absorption bands at 1427, 1367, 1334, 1029 cm^−1^ and 898 cm^−1^ belong to the stretching and bending vibrations of –CH_2_ and –CH, –OH and C–O bonds in cellulose [45,46].

Figure 2(2) shows the Fourier transform infrared spectrum recorded for polyurethane. The absorption band at 3323 cm^−1^ corresponds to NH stretching. The sharp peaks at 2858 cm^−1^ and 2925 cm^−1^ are associated with –CH_2_ stretching, while other modes of –CH_2_ vibrations are identified by the bands at 1448, 1406, 1334 and 1236 cm^−1^. In addition, the absorption band at 1631 cm^−1^ is associated with a C=O group in polyurethane. The group of NH bend vibrations is identified by the band at 1631 cm^−1^ [47].

The spectra of collagen [Figure 2(3)] shows the amide A band, associated with N–H stretching, at 3330 cm^−1^. The amide bands were observed at 1631, 1541 and 1334 cm^−1^, respectively. Polypeptide backbone C–O stretching vibration was found in the range of 1600–1700 cm^−1^. C–N stretching vibrations were noted at 1222 cm^−1^ [48].

**Figure 3:**
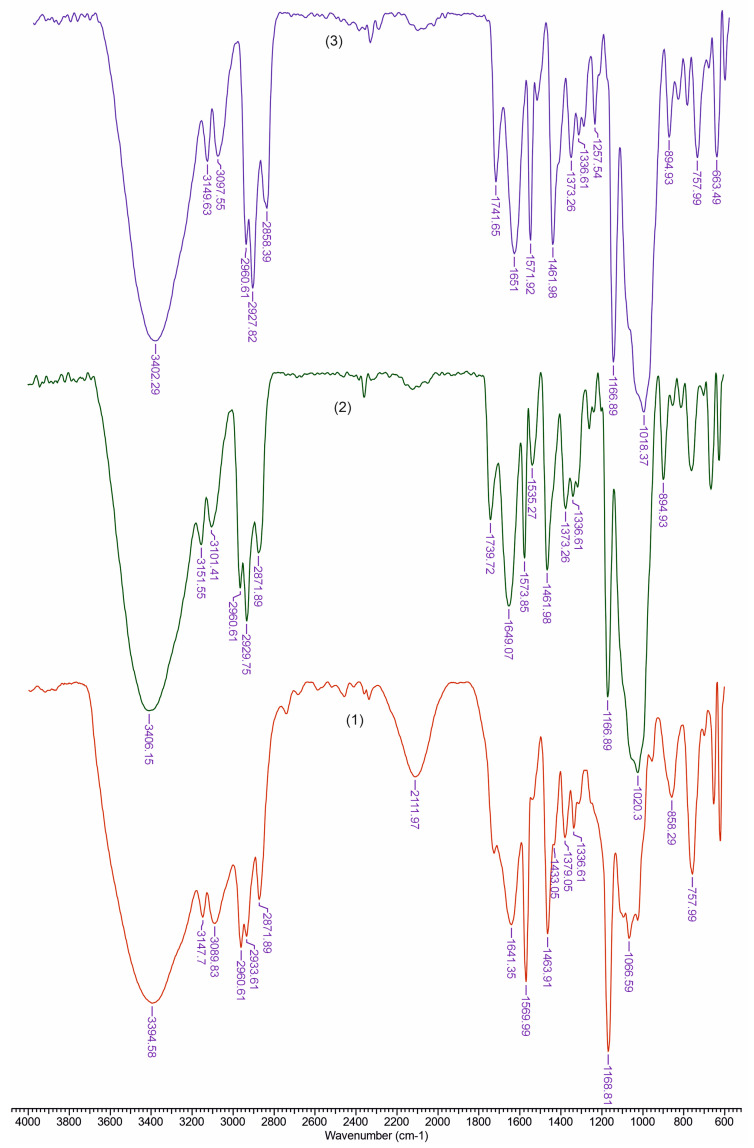
FTIR spectra of the obtained biomaterials: (**1**)—cellulose–collagen–polyurethane; (**2**)—TAN; (**3**)—LIP.

In Figure 3 are presented FTIR spectra for the obtained materials. Based on the recorded spectra, we can calculate a series of indices that reflect the degree of ordering and the total crystallinity, as well as the strength of the hydrogen bonds, for the studied materials.

The ratio between the heights of the bands at 1376 and 2902 cm^−1^ was proposed by Colomn and Carrillo [49] as the total crystalline index (TCI). The band at 1437 cm^−1^ is associated with the crystalline structure of cellulose, while the band at 899 cm^−1^ is assigned to the amorphous region in the cellulose. The ratio between the absorbance of the bands at 1437 and 899 cm^−1^ is used as a lateral order index (LOI). Considering the chain mobility and bond distance, the hydrogen bond intensity (HBI) of cellulose is closely related to the crystal system and the degree of intermolecular regularity—that is, crystallinity. The ratio of the absorbance bands at 3336 and 1336 cm^−1^ was used to study the cellulose sample’s HBI. The obtained results are displayed in Table 1. The TCI is proportional to the degree of crystallinity of cellulose, and LOI represents the ordered regions perpendicular to the chain direction in the cellulose.

The REF samples exhibited the highest TCI and lowest LOI, which implies the highest crystallinity degree and an increase in ordered regions perpendicular to the chain direction in cellulose. The data from Table 1 show that the LIP material presents the highest LOI and lowest value of TCI. It is possible that a lateral ordered cellulose structure was obtained in the cellulose-collagen-polyuretane matrix by the addition of lipoic acid. At the same time, the HBI value increased as compared to that of the matrix, which means that fewer available hydroxyl groups in the cellulose chain are able to interact by inter- and/or intramolecular hydrogen bonding.

On the contrary, the film comprised of tannin registered the highest value of HBI, suggesting strong interactions between the adjacent cellulose chains, resulting in a high level of cellulose chain packing, due to the numerous phenolic hydroxyl groups attached to the aromatic and heterocyclic rings. This also resulted in greater mechanical properties.

### 3.3. Mechanical Properties

**Figure 4:**
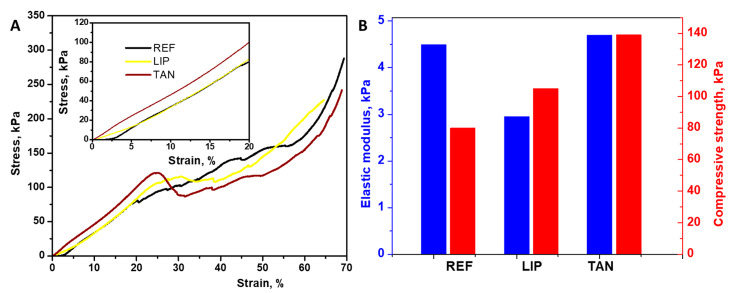
(**A**) Representative compressive stress-strain curves for REF, LIP and TAN at room temperature; (**B**) The values of the elastic modulus and compressive strength for the obtained biomaterials.

Compressive strength is an important parameter for the scaffolds used in tissue engineering. Consequently, uniaxial compression tests were applied to materials up to 70% strain, and the obtained stress-strain curves for all samples can be seen in Figure 4A. The tannin- and lipoic acid-containing biomaterials showed a typical linear stress-strain behavior at < 20% initial strain level (Figure 4A), demonstrating that the hydrogel blends changed from a relaxed state to a stressed state to store energy for resisting the compression stress [50]. As the strain level increased, the stress rapidly rose, and a fracture at about 20% strain level was first observed in the reference sample (Figure 4A), suggesting that the energy dissipation inside the network for this sample was not enough to resist the external force applied [51]. However, the reference material was found to be very elastic, with a value of elastic modulus of 4.5 kPa and a compressive strength of 80 kPa (Figure 4B). In general, the addition of filler causes the decrease of elasticity [52], but this influence was only observed for the sample containing lipoic acid (2.95 kPa, Figure 4B).

On the other hand, the addition of filler particles to the cellulose–collagen–polyurethane matrix induced a progressive increase of the compression strength, as well as the strain of the materials. When 10% of lipoic acid was added, the compression strength reached about 105 kPa, while the same amount of tannin increased compression strength up to 139 kPa. This means that the filler used can bear the stress effectively and increase the mechanical strength of the obtained materials.

The mechanical properties of polymer blends usually give an indication of possible interactions between their constituents [53]. The two-fold increase in the compressive strength of the materials containing tannin (TAN) indicate a possible interaction between the matrix and the filler within the blend via hydrogen bonding, as proved above by the highest HBI values (Table 1). The increase in mechanical properties, especially the rigidity of the samples containing tannin, could also be attributed to the decrease of the Brunauer–Emmett–Teller (BET) surface area and water sorption capacity (Table 2), because of the low dissipation energy inside the network. A low BET surface area indicates a reduced porosity of the sample, so the densification region which is normally observed for porous materials [54] was missing in our case, and thus the rigidity of the tannin-containing film increased (Figure 4B). The values of the elastic modulus of the materials prepared in this study are comparable to or even higher than those obtained for other cellulose-containing hydrogels. For example, Kalinoski & Shi [55] added lignin and/or xylan to cellulosic hydrogels, leading to values for the elastic modulus ranging from 20 to 105 kPa.

However, the values of the elastic modulus of our materials are weak compared to other cellulose-containing double network materials used for articular cartilage, which exhibited an elastic modulus of 322 kPa [56]. The results in the literature also suggest that by altering the ratios of xylan and lignin to cellulose, one can potentially fine-tune the mechanical properties of cellulosic hydrogels [57]. It is also possible to use chemical cross-linkers when preparing the physically cross-linked materials, which is especially effective in improving the matrix properties. The mechanical properties analysis provide evidence that cellulose–collagen–polyuretahane films with added lipoic acid and tannin become more resistant and less elastic. As our focus is to create physically cross-linked mechanical properties, materials using lipoic acid and tannin without chemical cross-linkers, or using chemical cross-linkers along with physical cross-linking methods, may warrant future study.

### 3.4. Water Adsorption Isotherms

**Figure 5:**
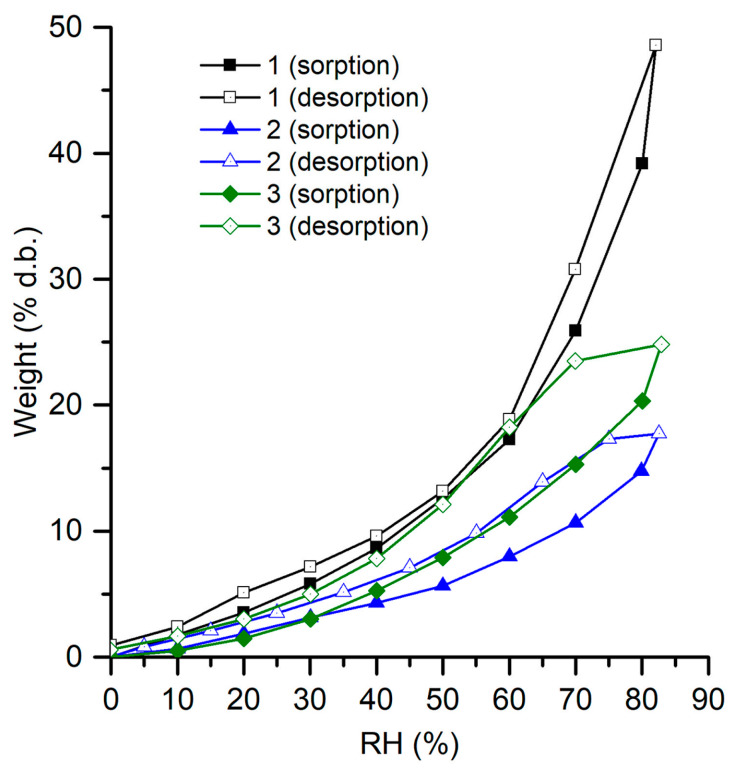
Sorption/desorption isotherms of the studied biomaterials (1—reference sample; 2—TAN sample; 3—LIP sample).

Adsorption and desorption isotherms express the dependence of equilibrium water content on materials and relative humidity. According to Figure 5, a hysteresis behavior is related to the water uptake capacity values of our materials.

The Brunauer–Emmett–Teller (BET) and Guggenheim–Anderson–de Boer (GAB) equations were used for the modelling of the sorption isotherms, but the BET model better fitted the experimental results as compared to the GAB model. This means that the transport phenomena are associated with the occurrence of monomolecular sorption range (I) and a multi-layer sorption (II). The data from Table 2 evidence a higher amount of water for the REF sample, which could also be correlated with the high elasticity already observed for this sample (Figure 4B). The addition of fillers to the polymeric matrix resulted in a significant decrease of the sorption capacity, the highest diminishing being recorded for the TAN sample. Other authors [58] reported that the adsorption of tannin on the cellulose surface is driven by the hydrophilic domains via H-bonding, and as a result, the hydrophobic domains of the tannin are exposed at the very top of the molecular surface.

### 3.5. Bio/Mucoadhesivity Properties

The developed materials present a surface layer possessing adhesive properties. Bio/mucoadhesive materials represent a promising tool to achieve site-specific drug delivery [59].

Adhesion of materials to the epithelial tissue is an important property for product safety, efficacy and quality. In Table 3, the results are presented from bioadhesion and mucoadhesion tests on cellulose dialysis membrane and porcine skin, respectively. The TAN addition induced a decrement of 33.56% of bioadhesion force, while lipoic acid presence did not influence this parameter.

The mucoadhesion test was performed in order to measure the ability of the films to adhere onto the porcine skin. The mucin present in the mucus surface layer of porcine skin is rich in cysteine (>10% of the amino acids) and therefore in the thiol groups which can lead the formation of many disulfide bonds (S–S) [60]. The thiol groups of mucins could interact with the hydroxyl or carboxyl groups of tannins, and these interactions could cause a better adhesion to the cell surface of porcine skin. An increment of 29.85% in mucoadhesiveness of TAN was recorded, and this could mean that the presence of tannin prolongs the material bioavailability, a fact confirmed by the drug release study. The LIP sample exhibited the highest mucoadhesiveness, mucoadhesive force being 1.85 times higher than that of the reference sample, since thiolate compounds are well-known as mucoadhesives [61].

The total work of the adhesion values is in good agreement with those of the adhesive forces.

### 3.6. Controlled Release of Active Compounds from Biomaterials

**Figure 6:**
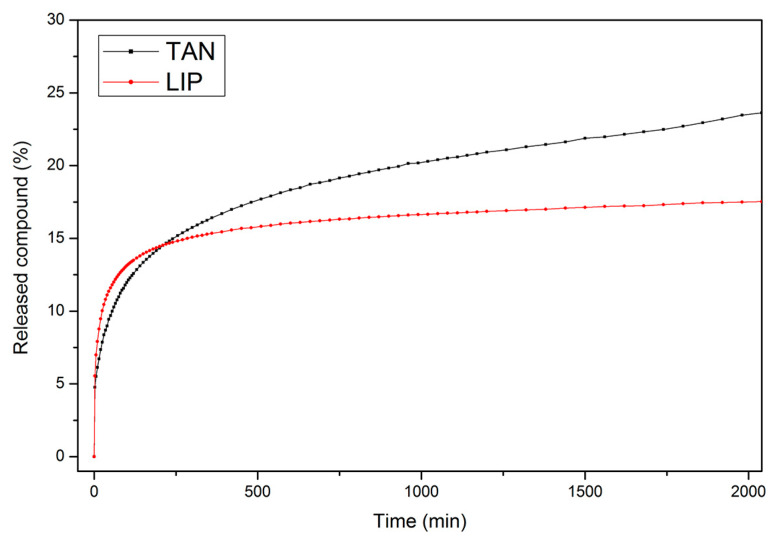
Release profiles of tannin and lipoic acid from the investigated samples.

The tannin and lipoic acid release profiles from the investigated materials were studied (Figure 6) to evaluate the potential delivery applications.

Comparing results presented in Figure 6 and Table 4, it seems that the highest amount of the active substance was released from the TAN material, which is in a good agreement with the findings regarding adhesive properties (Table 3). For both samples, the correlation coefficient (*R*^2^) was rather high (0.998), and describes the first-order kinetic model. The transport constants (*k*) and transport exponents (*n*) were determined from the obtained data. The k value was higher for the LIP sample as compared to the TAN sample. The value of *n* was less than 0.3 for both samples, and this corresponds to a Fickian diffusion, suggesting that the release mechanisms of both fillers were related to the physical diffusion/filler dissolution interaction of electrostatic forces or hydrogen bonds [62].

### 3.7. Antiradical Activity

**Figure 7:**
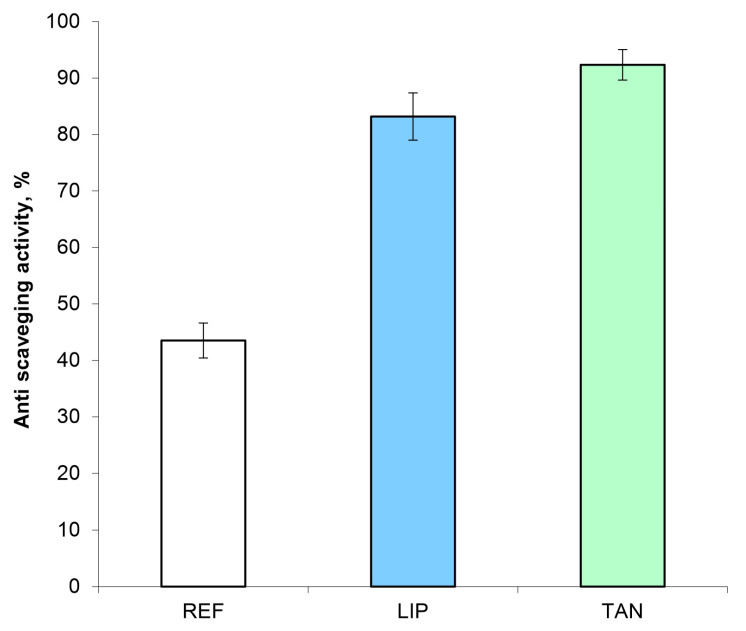
Antiradical activity of the obtained biomaterials.

It is well known that free radicals are able to induce oxidative stress in biomolecules, thus causing a wide range of degenerative diseases. The free radical scavenging activity of TAN and LIP materials confirms their potential therapeutic value in protecting against oxidative injury. The results from Figure 7 show that the materials are able to scavenge the free radicals, so reducing the oxidative stress. The material comprised of tannin as the bioactive compound presented the highest ability to scavenge free radicals [63]. This can be explained by the hydrogen-donating phenolic hydroxyl groups attached to the aromatic and heterocyclic rings, which impart good antioxidant activity to the TAN film [59]. It is worth mentioning that free tannin and tannin in the cellulose–collagen–polyurethane matrix exhibited almost the same radical scavenging activity (92.34% and 92.03%). The LIP material exhibited scavenging activity of DPPH radical (83.18%) lower than free lipoic acid (87.05%), probably due to the interactions between the components of the material.

### 3.8. Materials’ Morphology

**Figure 8:**
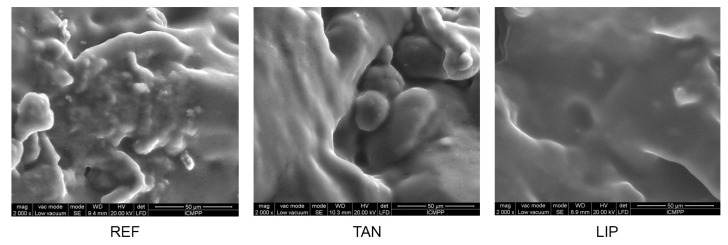
Images of materials.

Investigating the SEM images (Figure 8) can give different valuable information about film morphology. When filler was added into the matrix, its surface tended to become smoother. The absence of voids, as well as the presence of some discrete micro-domains, suggests the development of hydrogen bond networks between matrix components and fillers, confirmed by the HBI value (Table 1). It also suggests that the compatibility and interfacial strength between the filler particles and cellulose–collagen–polyurethane matrix is improved.

## 4. Conclusions

A new biomaterial comprising cellulose, collagen and polyurethane was obtained by dissolution in butyl methylimidazole chloride. Other formulations containing lipoic acid and tannin were developed and analyzed. The addition of filler particles to the cellulose–collagen–polyurethane matrix induced a progressive increase of the compression strength, as well as the strain of the materials, which means that the filler used can bear stress effectively and increase the mechanical strength of the obtained materials. A hysteresis behavior is related to the water uptake capacity values of the materials, while when filler was added into the matrix, its surface tended to become smoother.

Perhaps the most important implication of this study is associated with the hypothesis that the fillers’ addition to the polymeric matrix induces improved biological properties, a fact confirmed by the increasing of the mucoadhesiveness, as well as of the anti-scavenging activity. The in vitro release of the used fillers is described by Korsmeyer–Peppas model.

In summary, the obtained results confirm that the prepared materials could be promising carriers for controlled release of TAN and LIP, with potential medical and cosmetic applications. For perspective, we are already considering the incorporation of active principles with anti-cellulite action (for cosmetic applications, obviously) into the polymer matrix, that will prove it has mechanical strength, elasticity and bioadhesiveness.

## Figures and Tables

**Table 1:** Infrared total crystallinity (TCI), hydrogen bond intensity (HBI) and lateral order index (LOI) for the obtained biomaterials.

	TCI (A_1376_/A_2902_)	LOI (A_1437_/A_899_)	HBI (A_3336_/A_1336_)
REF	0.492	1.533	3.459
TAN	0.447	2.132	4.368
LIP	0.406	2.250	4.177

**Table 2:** Dynamic vapors sorption (DVS) parameters for the obtained biomaterials.

Sample	Sorption Capacity % d.b.	BET Data	
		Area m^2^ × g^−1^	Monolayer g × g^−1^
REF	48.5	331.062	0.094
TAN	17.7	151.100	0.043
LIP	24.8	758.750	0.216

**Table 3:** Adhesive properties of the studied materials.

Sample	Bioadhesion Test		Muchoadhesion Test	
	Adhesion Force (*n*)	Total Work of Adhesion (*n* × *s*)	Adhesion Force (*n*)	Total Work of Adhesion (*n* × *s*)
REF	0.143 ± 0.00205	0.025 ± 0.00286	0.067 ± 0.00339	0.0099 ± 0.00033
TAN	0.095 ± 0.00205	0.020 ± 0.0017	0.087 ± 0.00449	0.0204 ± 0.00041
LIP	0.142 ± 0.00368	0.031 ± 0.00163	0.124 ± 0.0033	0.0317 ± 0.00057

**Table 4:** Kinetic parameters of the fillers released from investigated samples.

Samples	*n*	*R* ^2^_n_	*k*, min^−n^	*R* ^2^_k_
TAN	0.294	0.998	0.0308	0.999
LIP	0.235	0.998	0.0500	0.999

*n* = release exponent, *k* = release rate constant, *R*^2^_n_ and *R*^2^_k_ = correlation coefficient. Corresponding to the slope obtained for determination of *n* and *k*.
